# Self-control and SAT outcomes: Evidence from two national field studies

**DOI:** 10.1371/journal.pone.0274380

**Published:** 2022-09-28

**Authors:** Chayce R. Baldwin, Kyla Haimovitz, Priya Shankar, Robert Gallop, David Yeager, James J. Gross, Angela L. Duckworth

**Affiliations:** 1 Department of Psychology, University of Michigan, Ann Arbor, Michigan, United States of America; 2 Department of Psychology, University of Pennsylvania, Philadelphia, Pennsylvania, United States of America; 3 Department of Mathematics, West Chester University of Pennsylvania, West Chester, Pennsylvania, United States of America; 4 Department of Psychology, University of Texas, Austin, Texas, United States of America; 5 Department of Psychology, Stanford University, Stanford, California, United States of America; Carnegie Mellon University, UNITED STATES

## Abstract

Self-control is often thought to be synonymous with willpower, defined as the direct modulation of impulses in order to do what is best in the long-run. However, research has also identified more strategic approaches to self-control that require less effort than willpower. To date, field research is lacking that compares the efficacy of willpower to strategic self-control for consequential and objectively measured real-world outcomes. In collaboration with the College Board, we surveyed two national samples of high school students about how they motivated themselves to study for the SAT college admission exam. In Study 1 (*N* = 5,563), compared to willpower, strategic self-control predicted more hours of SAT practice and higher SAT scores, even when controlling for prior PSAT scores. Additionally, the more self-control strategies students deployed, the higher their SAT scores. Consistent with dose-response curves in other domains, there were positive albeit diminishing marginal returns to additional strategies. Mediation analyses suggest that the benefits of self-control strategies to SAT scores was fully explained by increased practice time. These results were confirmed in Study 2, a preregistered replication with *N* = 14,259 high school students. Compared to willpower, strategic self-control may be especially beneficial in facilitating the pursuit of goals in high-stakes, real-world situations.

## Introduction

In popular parlance, self-control is typically equated with brute-force efforts to control one’s behavior [1]. But direct regulation of conflicting impulses is only one way to achieve valued goals [2,3]. Specifically, theorists have differentiated between *strategic* and *willpower* approaches to enacting self-control [4–7]. Strategic self-control entails the use of situational and cognitive strategies to voluntarily align thoughts, feelings, and actions with enduringly valued goals despite momentarily more alluring alternatives [4]. For example, strategic self-control can take the form of keeping temptations out of sight, reminding oneself of the importance of a valued goal, or monitoring goal progress. Willpower, in contrast, entails direct, in-the-moment regulation of ongoing behavior—“just” saying no to temptations, for example, or “just” forcing oneself to get started on a valued goal.

Strategic self-control is theorized to be more efficient and effective than willpower [3,4], which is typically experienced as effortful, aversive, and fatiguing [8–10]. Consistent with this expectation, directly modulating responses is difficult to sustain [11,12]—but laboratory studies have demonstrated the efficacy of an array of self-control strategies. For example, in the preschool delay of gratification paradigm, children encouraged to think happy thoughts waited longer for a preferred treat than children given no instruction [13]. So did children whose view of treats was obscured by an overturned tray [14] or who, when treats were visible, spontaneously averted their gaze [15]. Likewise, when prompted to use psychological distancing (i.e., the approach of referring to themselves in the third person) when recalling an upsetting memory, both adults and children were better able to regulate their negative emotions [16–18].

In addition to differentiating between strategic self-control and willpower, laboratory research has shown the benefits of “polyregulation”: the deployment of more than one self-control strategy toward the same goal [19]. For example, adults who spontaneously employed more than one self-control strategy while watching a disgust-eliciting film clip were better able to regulate their emotions [20]. Experience Sampling Methodology (ESM) studies have also demonstrated the value of polyregulation. For example, Williamson and Wilkowski [21] asked a total of 179 undergraduates in two separate samples to identify three different personal goals and then, during the following week, to periodically report on their momentary use of self-control strategies. Four self-control strategies were each consistently and independently associated with goal progress. In contrast, the benefits of using willpower were inconsistent—showing sizeable effects in one sample but not in the other. Similarly, Milyavskaya et al. [22] asked 197 volunteers, mostly undergraduates, to spend a week reporting on their momentary temptations and, each time, to indicate which, if any, of eight different self-control strategies they used to address them. Each of the measured self-control strategies was independently associated with resisting temptations, and the more self-control strategies that were used, the greater the likelihood of success. However, in contrast to Williamson and Wilkowski [21], strategic self-control and willpower were equally effective.

In sum, prior research suggests that self-control can take the form of either strategic self-control or willpower, and that deploying a variety of self-control strategies can be beneficial. However, there has been no direct comparison between the relative efficacy of strategic self-control and willpower in high-stakes settings with objectively measured outcomes. Similarly, there has been no direct investigation of the advantages of polyregulation in such settings, particularly with the very large samples needed to explore the possibility of non-linear effects (e.g., declining marginal benefits) of polyregulation on behavior and outcomes. While preliminary research has suggested that polyregulation may be efficacious [22], research has not yet investigated the effects of using more than a small number of strategies at once. Additionally, past theory posits that, in some situations, too much regulation could have deleterious effects [19,23], leaving open questions of nonlinear effects of polyregulation.

In the current investigation, we partnered with the College Board to conduct two national field studies on strategic self-control and willpower in a real-world, high-stakes setting: the SAT college entrance exam. Research has shown that self-control predicts academic achievement [24,25] and goal-directed day-to-day academic behavior, including studying [26,27]. Given this past empirical and theoretical research on self-control, as well as clear documentation of the value of practice time [28], we hypothesized that compared to willpower, strategic self-control would better predict hours of SAT practice and performance. In addition, we anticipated benefits of polyregulation, expecting that using a greater variety of self-control strategies to self-regulate study behavior would predict cumulative SAT practice time, which in turn would predict SAT scores. In Study 1 (*N* = 5,563), high school students reported on their use of strategic self-control and willpower when motivating themselves to study for the SAT. Study 2 was a preregistered conceptual replication in a larger sample (*N* = 14,259) and used a refined survey instrument. When creating materials for both studies, we adhered to principles of user-centered design, iteratively prototyping materials in dialogue with separate samples of adolescents preparing for the SAT [see 29].

## Study 1: Self-control, studying, and SAT performance

In Study 1, high school students reported how they motivated themselves to study for the SAT and how much they practiced. These responses were then linked to official records of SAT scores, demographics, and, as a baseline indicator of academic achievement, prior PSAT scores.

### Method

#### Participants

A total of *N* = 5,937 high school students who took the SAT in August 2017 completed the study survey. Because all analyses controlled for PSAT scores and demographic variables, we retained only those for whom there was complete data on each of these measures, resulting in a final sample of *N* = 5,563 students. The students were 51% White, 20% Asian, 13% Hispanic, 9% Black, and 4% other racial-ethnic backgrounds; 70% were female. The large majority, over 99%, were from the United States. As an indication of lower family socioeconomic status, 13% of students received fee waivers for taking the SAT.

#### Procedure

The College Board invited students who took the SAT in August 2017 to complete an optional online survey, which was available for two weeks after the SAT was administered but before students received their scores. Sample size was determined by the existing data collection procedures of the College Board. This study was approved by the Institutional Review Board at the University of Pennsylvania. The College Board administered the surveys using their own existing consent processes.

#### Measures

*Strategic self-control*. As shown in **Table 1**, students indicated whether they used any of a dozen different self-control strategies (e.g., “I chose to study in places that were easier to focus”) to help themselves practice for the SAT. These items were informed by prior research on self-control strategies, but primarily developed through qualitative pilot work involving a separate sample of high school students on how they motivated themselves to practice.

**Table 1:** Study 1 individual self-control approaches.

Self-Control Strategy	% Used
I reminded myself why I was studying in the first place.^a^	59
I chose to study in places that were easier to focus.^a^	49
I set up a place to study that was free of distractions.^a^	40
I disabled my phone while I practiced.^a^	31
I set a concrete study schedule.^a^	24
I tracked how often I studied.^a^	24
I told my study goals to someone who cared about me.^a^	21
I reminded myself that frustration is a sign of learning.^a^	19
I thought about the skills I was building for later in life.^a^	16
I made a visual reminder of why I was studying.^a^	12
I tried to turn studying into a game.^a^	8
I made a study plan with a friend.^a^	6
I just forced myself to do it.^b^	72

^a^ Strategic self-control items.

^b^ Willpower item.

We created two variables: A binary variable was coded 1 if students indicated that they used any of the self-control strategies listed and 0 if they endorsed none. A count variable ranging from 0 to 12 indicated the number of self-control strategies endorsed.

*Willpower*. As shown in **Table 1**, students indicated the use of willpower to practice for the SAT by endorsing the single item: “I just forced myself to do it”. We coded willpower as 1 if they indicated they “just forced” themselves and 0 if they did not. This item is similar to measures used in recent research [21,22].

*Practice time*. Using a slider scale from 0 to 30 days, students indicated “how many days did you practice for the SAT in the last month?” Likewise, using a slider scale from 0 to 180 minutes, students indicated, “On the days you practiced, about how many minutes did you practice per day?” We multiplied these two values to estimate total practice time. Students reported practicing an average of 16 hours (*M* = 16.28, *SD* = 19.47). Because this composite variable was positively-skewed, we used log-transformed values in all analyses. There were no outliers (more than three standard deviations above or below the mean) in reported practice time.

*SAT scores*, *PSAT scores*, *and demographics*. For each student, the College Board provided the August 2017 SAT scores, as well as covariates that have demonstrated significant associations with SAT performance: PSAT scores (the Pre-SAT, usually taken by 10^th^ and 11^th^ graders in the U.S.), gender, ethnicity, and fee waiver status [see 30]. Outliers in SAT scores (more than three standard deviations above or below the mean; *n* = 6) were excluded from analyses. The average student in our sample scored in the 90th national percentile on the PSAT (*M* = 1,174, *SD* = 180) and in the 81st national percentile on the SAT (*M* = 1,248, *SD* = 179).

#### Analytic strategy

In all regression models, in order to isolate the effect of strategic self-control and willpower among students of comparable backgrounds, we controlled for well-known predictors of SAT performance: prior PSAT scores and the demographic covariates of gender, ethnicity, and fee waiver status.

We assessed the benefits of strategic self-control in two ways. First, we fit simultaneous multiple regression models predicting practice time and SAT scores, respectively, from the binary predictors of the willpower and the use of any self-control strategy, and analyzed linear contrasts between these effects. Second, we examined the dose-response relationship between these outcomes and the number of self-control strategies. Specifically, we fit regression models predicting practice time and SAT scores, respectively, from the number of self-control strategies used. To identify curvilinearity, we also fit models including a quadratic term of number of self-control strategies squared predicting practice time and SAT scores. Finally, we fit a bootstrapped and bias-corrected mediation model to assess whether practice time accounted for the relationship between the number of self-control strategies used and SAT scores [31]. In addition to reporting unstandardized regression coefficients, we report standardized regression coefficients to compare coefficients across SAT practice and performance. For models with binary predictors, the coefficients are partially standardized, such that only the dependent variable, and not the binary predictors, is standardized.

In S1 Appendix, we provide coefficients for all predictors in regression models presented in the main text and demonstrate the robustness of these results in a series of alternative models that reveal the same pattern of results.

### Results and discussion

To motivate themselves to practice during the month prior to the SAT, 83% of students recalled using at least one self-control strategy, and 72% of students recalled using willpower (see S1 Appendix for individual strategies’ effects on outcomes). The tendency to use strategic self-control was positively related to the tendency to use willpower (*χ*^2^ (5563) = 330.99, *p* < .001, Φ = .24). Sixty-four percent of students reported using both willpower and at least one self-control strategy. Bivariate correlations and descriptive statistics for all variables are provided in S1 Appendix.

Compared to using willpower, using at least one self-control strategy predicted practicing more and earning higher SAT scores: When both binary predictors were included in a simultaneous multiple regression model that also controlled PSAT scores, gender, ethnicity, and fee waiver status, using at least one self-control strategy predicted practice time (*B* = 1.02, *β* = 1.18, *p* < .001, 95% CI [0.96, 1.07]) better than willpower (*B* = 0.28, *β* = .32, *p* < .001, 95% CI [0.23, 0.33]; linear contrast: *B* = 0.74, 95% CI [0.66, 0.82], *t*(5023) = 18.11, *p* < .001). Likewise, using at least one self-control strategy also predicted SAT scores (*B* = 19.26, *β* = .11, *p* < .001. 95% CI [13.61, 24.91]) better than willpower (*B* = 7.42, *β* = .04, *p* < .01, 95% CI [2.64, 12.19]; linear contrast: *B* = 11.84, 95% CI [3.62, 20.07], *t*(5540) *=* 2.82, *p* < .01).

**Fig 1:**
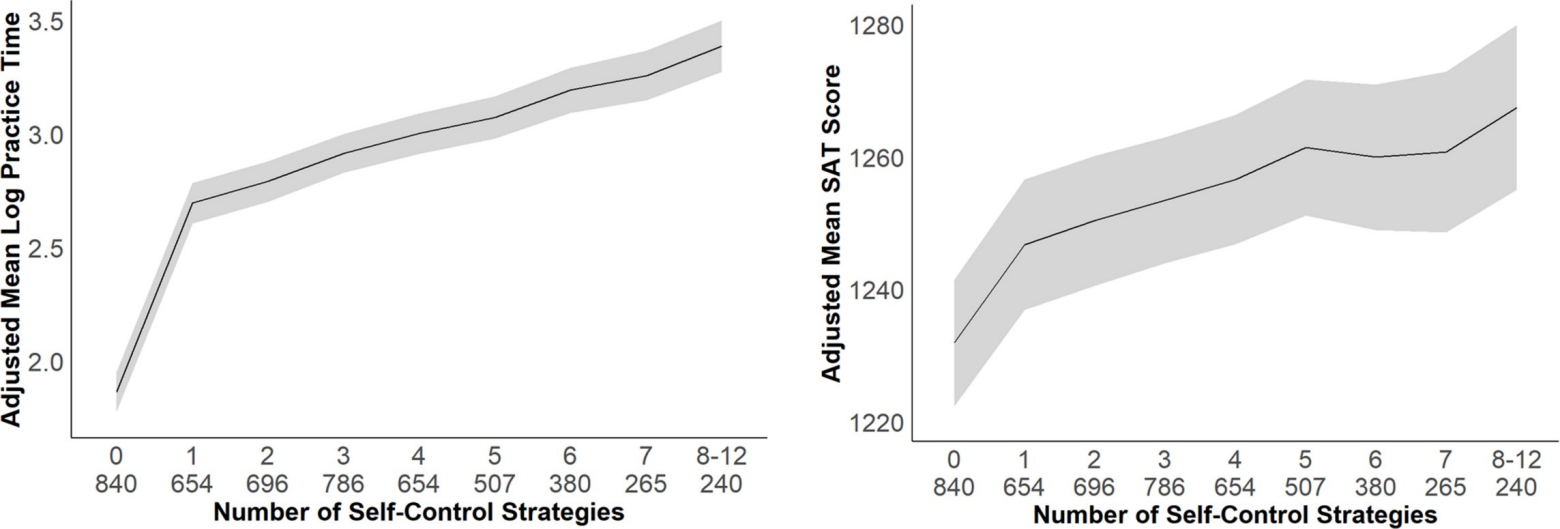
Number of self-control strategies used predicting practice time and SAT scores for Study 1. Study 1 practice time (panel *a*) and SAT score (panel *b*) as a function of the number of self-control strategies used. Means are adjusted for covariates: PSAT scores, gender, ethnicity, and fee waiver status. Shading indicates 95% confidence intervals.

As shown in **Fig 1**, there was a positive linear relationship between the number of self-control strategies used and total practice time, but adding a quadratic term revealed there was also a decelerating dose-response relationship. Specifically, the more self-control strategies students used, the more time they spent practicing (*B* = 0.34, *β* = .95, *p* < .001, 95% CI [0.31, 0.37]), but the marginal benefit of additional strategies was smaller (*B* = -0.02, *β* = -.54, *p* < .001, 95% CI [-0.03, -0.02]). Likewise, the number of self-control strategies students used predicted higher SAT scores (*B* = 7.33, *β* = .10, *p* < .001, 95% CI [4.87, 9.79]), but the marginal benefit of additional approaches was slightly smaller (*B* = -0.46, *β* = -.05, *p* = .003, 95% CI [-0.76, -0.15]). Results remain the same whether we treat the predictor as a linear or curvilinear predictor (see S1 Appendix).

**Fig 2:**
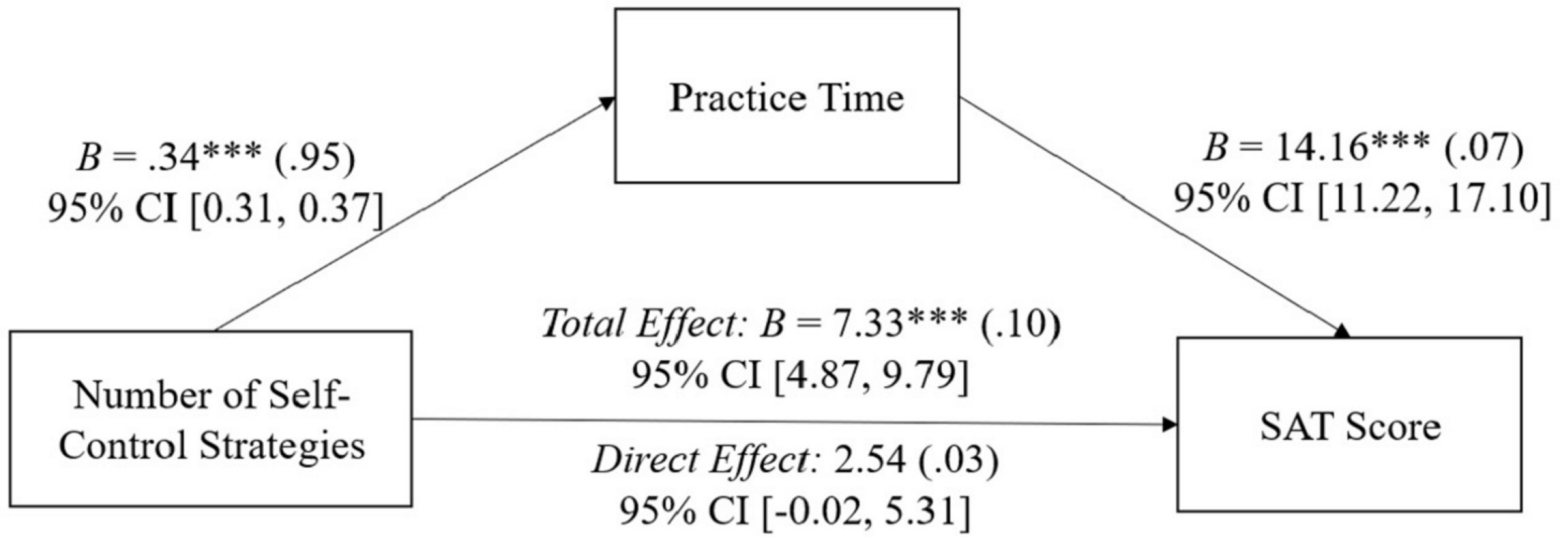
Study 1 practice time mediating the relationship between number of self-control strategies and SAT scores. Study 1 practice time mediates the relationship between the number of self-control strategies and SAT scores. *B* represents the unstandardized beta coefficient (standardized beta coefficients in parentheses). Covariates include the squared strategic self-control term, students’ PSAT scores, gender, fee waiver status, and ethnicity. ***p < .001.

As shown in **Fig 2**, the benefits of strategic self-control were mediated by practice time. When controlling for practice time, the effect of the number of self-control strategies used on SAT score was reduced (*B* = 2.54, *β* = .03, *p* = .062, 95% CI [-0.13, 5.20]), as was the marginal benefit of additional strategies (i.e., the quadratic effect; *B* = -0.13, *β* = -.01, *p* = .432, 95% CI [-0.44, 0.19]), with a significant indirect effect of *B* = 4.79 confirmed with a 10,000 bootstrapped resampling with 95% bias-corrected confidence interval from 3.74 to 5.87.

In sum, compared to using willpower, using at least one self-control strategy was a stronger predictor of practice time and SAT scores when controlling for prior achievement and demographic covariates. In addition, there was a positive but decelerating dose-response curve between the number of self-control strategies and practice time, which in turn mediated the relationship between self-control strategies and SAT scores.

## Study 2: Preregistered replication

In Study 2, we conducted a preregistered conceptual replication of Study 1 (https://osf.io/4n7md/) with a larger sample of high school students who completed a refined set of questionnaire items assessing strategic self-control and willpower.

### Method

#### Participants

A total of *N* = 15,193 high school students who took the SAT in August 2018 completed the study survey. As in Study 1, we retained only those for whom there was complete data on each of the covariates used in analyses, resulting in a final sample of *N* = 14,259 students. The students were 41% White, 27% Asian, 17% Hispanic, 8% Black, and 5% other racial-ethnic backgrounds; 64% were female. The large majority, 99%, were from the United States, and 8% received fee waivers for taking the SAT. Sample size was again determined by the existing data collection procedures of the College Board.

#### Procedure

We followed the same procedure as in Study 1, with two unanticipated exceptions disclosed to us by the College Board after data collection was complete. First, to reduce survey burden, the College Board showed each student a randomly selected subset of 5 of 17 possible items assessing strategic self-control and willpower. Second, the College Board changed the presentation for these items from the checklist used in Study 1 to individual questions with three possible responses: “yes”, “no”, and “not sure.” For consistency with Study 1, we considered “yes” as an endorsement of that item. Additionally, while all students saw at least two strategic self-control items, showing students a random subset of items led to 35% of students not seeing any willpower items. These students were excluded from analyses that included the measure of willpower. This study was approved by the Institutional Review Board at the University of Pennsylvania. The College Board administered the surveys using their own existing consent processes.

#### Measures

Prior to Study 2, we worked with Character Lab Research Network to conduct one-on-one interviews with more than two dozen high school students about how they and their peers motivated themselves to practice for the SAT. One of the authors (K.H.) also shadowed three high school juniors on separate days for a more in-depth understanding of how SAT practice fit into students’ daily lives. These interactions informed the refinement of the checklist of strategic self-control and willpower items for Study 2.

*Strategic self-control*. As shown in **Table 2**, the pool of 14 self-control strategies included eight items identical to those used in Study 1. To simplify language and clarify meaning, we revised three items used in Study 1 (e.g., “I disabled my phone while I practiced” to “I turned off or hid my phone while I practiced”). Likewise, we dropped the least frequently endorsed item in Study 1: “I made a study plan with a friend.” Finally, we added three new strategies: “I turned my attention away from distractions while I studied,” “I thought of the ways that distractions from practicing would be harmful,” and “I changed the way I was thinking about the SAT to make it easier to study.”

**Table 2:** Study 2 individual self-control approaches.

Self-Control Strategy	% Used
I chose to study in places that were easier to focus.^a^	77
I reminded myself why I was studying in the first place.^a^	76
I turned my attention away from distractions while I studied.^a^	73
I set up a place to study that was free of distractions.^a^	65
I turned off or hid my phone while I practiced.^a^	54
I changed the way I was thinking about the SAT to make it easier to study.^a^	53
I thought of the ways that distractions from practicing would be harmful.^a^	51
I tracked how often I studied.^a^	46
I told my study goals to someone who cared about me.^a^	44
I set reminders to practice somewhere I would see them.^a^	43
I set a concrete study schedule somewhere I would see regularly.^a^	40
I thought about the skills I was building for later in life.^a^	38
I reminded myself that frustration is a sign of learning.^a^	35
I tried to turn studying into a game.^a^	17
I didn’t do anything in particular, I just willed myself to not get distracted.^b^	56
I didn’t use different ways to practice, I just forced myself to do it.^b^	50
I didn’t use strategies, I just gritted my teeth and tried hard to study.^b^	32

^a^ Strategic self-control items.

^b^ Willpower items.

Consistent with Study 1 and our preregistration, we created two variables: A binary variable was coded 1 if students indicated that they used any of the strategies listed and 0 if they endorsed none. A count variable ranging from 0 to 5 indicated the number of self-control strategies endorsed.

*Willpower*. Qualitative interviews suggested that the item used to assess willpower in Study 1 (“I just forced myself to do it”) may have been interpreted by some students as “I studied independently and not because my parents or teachers forced me to do so.” Additionally, a single item for willpower, as used in Study 1, may not have adequate measurement precision. As shown in Table 2, to increase measurement precision and more strongly contrast strategic self-control and willpower, we developed three items in Study 2 that more explicitly specified the use of willpower. Further, in order to test whether our effects in Study 2 are a result of higher measurement precision in strategic self-control than willpower, we re-analyzed the main models for the subset of students that saw at least two willpower items (and thus at most three strategic self-control items), to assess whether the effects hold when participants see a similar number of items for each measurement. Effect sizes observed in these models were consistent with results from the full sample, suggesting that our observed effects were not due to varying levels of measurement precision across measures (see S1 Appendix for details).

Consistent with Study 1, we coded willpower as 1 if students endorsed any of these items and 0 if they did not.

*Practice time*. We measured practice time in the same way as Study 1, except that the question about how many minutes they practiced on study days ranged from 1 to 180 minutes per day. On average, students reported practicing 19 hours (*M* = 19.00, *SD* = 21.06) in the month prior to the SAT. Because this composite variable was positively skewed, we used log-transformed values in all analyses. Additionally, outliers in reported practice time (more than three standard deviations above or below the mean; *n =* 388) were excluded from analyses.

*SAT and PSAT scores and demographics*. As in Study 1, we obtained test scores and student demographics from the College Board. The average student in our sample scored in the 90th national percentile on the PSAT (*M* = 1,165, *SD* = 169) and in the 80th national percentile on the SAT (*M* = 1,255, *SD* = 179). Outliers in SAT scores (more than three standard deviations above or below the mean; *n* = 12) were excluded from analyses.

#### Analytic strategy

As in Study 1, and as specified in our preregistration, we controlled for prior PSAT scores and the demographic covariates of gender, ethnicity, and fee waiver status in all regression models.

We followed the same analytic strategy as in Study 1. In our preregistered plan, we anticipated all analyses with one exception: We failed to specify including a quadratic term to account for the possibility of non-linearity in the relationship between the number of self-control strategies used and the outcomes of practice time and SAT scores, respectively. Preliminary analyses indicated it was in fact appropriate to include a quadratic term, and so we do so the analyses presented below. In S1 Appendix, we show that the results are similar whether or not this quadratic term is included, and additionally present alternate models to the linear contrasts comparing the effects of strategic self-control and willpower that likewise support the robustness of the results.

### Results and discussion

Similar to Study 1, 86% of students in Study 2 recalled using at least one self-control strategy in the month prior to the SAT. However, as expected, revising willpower items to emphasize an effortful, direct approach to self-control resulted in a smaller proportion of students (51%) reporting the use of willpower to motivate themselves to practice in the month prior to the SAT. Relatedly, the tendency to use willpower was now inversely related with the tendency to use strategic self-control (*χ*^2^ (9269) = 14.54, *p* < .001, Φ = -.04). Additionally, in contrast to Study 1, only 42% of students endorsed both at least one willpower item and one strategic self-control item. Bivariate correlations and descriptive statistics for all items are provided in S1 Appendix.

Consistent with Study 1, using at least one self-control strategy was a stronger predictor than willpower of spending more time practicing as well as earning higher SAT scores: When both binary predictors were included in a simultaneous multiple regression model that also controlled PSAT scores, gender, ethnicity, and fee waiver status, using a self-control strategy predicted practice time (*B* = 0.32, *β* = .20, *p* < .001, 95% CI [0.29, 0.35]) better than willpower (*B* = -0.09, *β* = -.08, *p* < .001, 95% CI [-0.11, -0.07]); linear contrast: *B* = 0.41, 95% CI [0.37, 0.44], *t*(8799) = 22.03, *p* < .001). Likewise, using a self-control strategy also predicted SAT scores (*B* = 7.13, *β* = .02, *p* = .001, 95% CI [2.73, 11.52]) better than willpower (*B* = -4.88, *β* = -.01, *p* = .003, 95% CI [-8.13, -1.63]; linear contrast: *B* = 12.01, 95% CI [6.66, 17.37], *t*(9184) = 4.40, *p* < .001).

**Fig 3:**
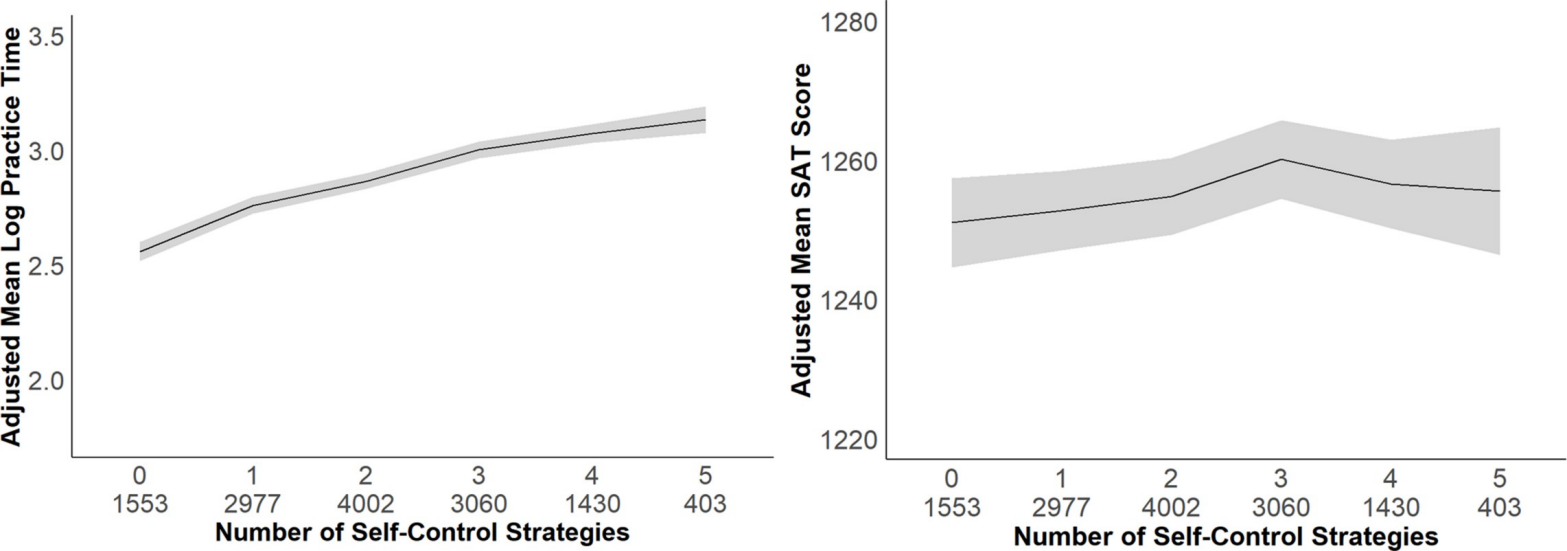
Number of self-control strategies used predicting practice time and SAT scores for Study 2. Study 2 practice time (panel *a*) and SAT score (panel *b*) as a function of number of self-control strategies used. Means are adjusted for covariates: PSAT scores, gender, ethnicity, and fee waiver status. Shading indicates 95% confidence intervals.

As shown in **Fig 3**, the decelerating dose-response relationship between the number of self-control strategies used and total practice time was similar to what was observed in Study 1. Specifically, the greater the number of self-control strategies students used, the more time they spent practicing (*B* = 0.19, *β* = .34, *p* < .001, 95% CI [0.16, 0.21]), but the marginal benefit of additional strategies was smaller (*B* = -0.01, *β* = -.12, *p* < .001, 95% CI [-0.02, -0.01]). The number of self-control strategies students used predicted higher SAT scores (*B* = 4.30, *β* = .03, *p* = .01, 95% CI [1.01, 7.59]), with declining marginal returns to additional endorsed strategies and, in contrast to Study 1, a slight, but nonsignificant concave relationship, in which SAT scores declined slightly beyond the endorsement of 3 of 5 presented strategies (*B* = -0.57, *β* = -.02, *p* = .129, 95% CI [-1.30, 0.16]).

**Fig 4:**
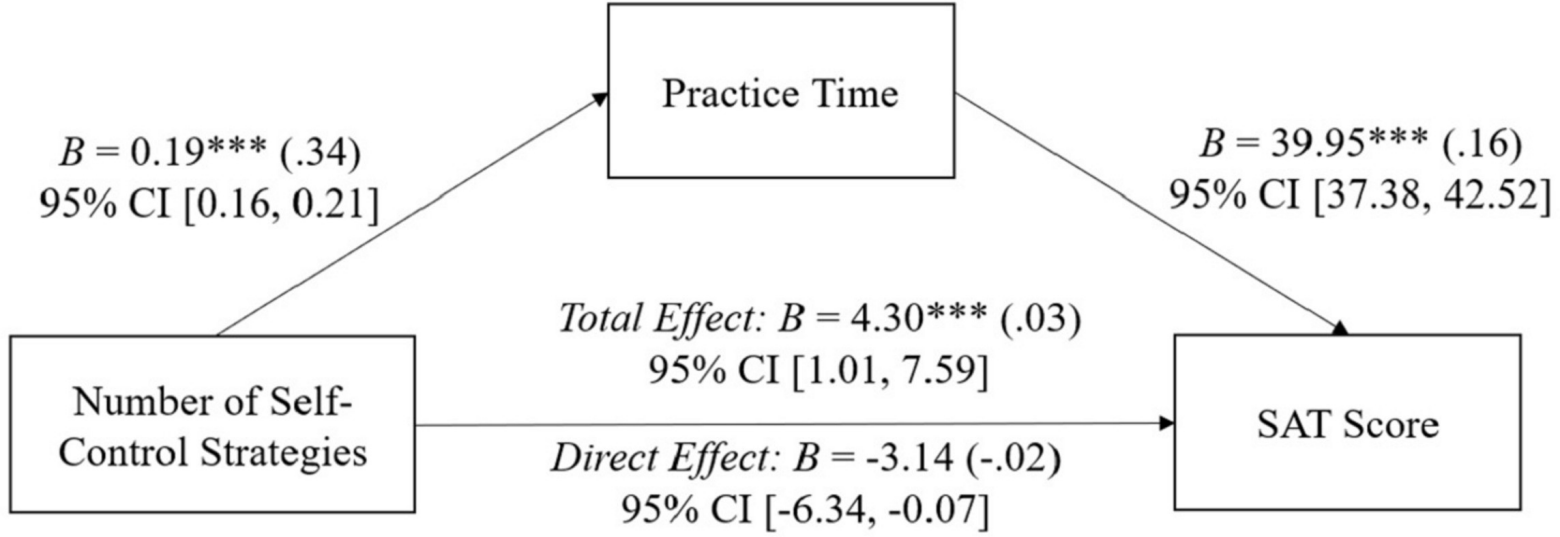
Study 2 practice time mediating the relationship between number of self-control strategies and SAT scores. Study 2 practice time mediates the relationship between the number of self-control strategies and SAT scores. *B* represents the unstandardized beta coefficient (standardized beta coefficients in parentheses). Covariates include the squared strategic self-control term, students’ PSAT scores, gender, fee waiver status, and ethnicity. *** *p* < .001.

As shown in **Fig 4**, the benefits of strategic self-control were again mediated by practice time: When controlling for practice time, the effect of the number of self-control strategies used on SAT score was reduced, and in fact became negative, indicating a suppression effect (*B* = -3.14, *β* = -.02, *p* = .055, 95% CI [-6.34, 0.07]), and the quadratic term became nonsignificant (*B* = 0.03, *β* = .001, *p* = .931, 95% CI [-0.68, 0.74]). The significant indirect effect of *B* = 7.44 was confirmed through a 10,000 bootstrapped resampling with 95% bias corrected confidence interval from 6.42 to 8.49.

In sum, Study 2 replicated the pattern of results observed in Study 1. Compared to using willpower, using at least one self-control strategy was a stronger predictor of practice time and SAT scores when controlling for prior achievement and demographic covariates. In addition, we observed a decelerating dose-response curve between the number of self-control strategies and practice time, which in turn mediated the relationship between self-control strategies and SAT scores.

## Mega-analysis of Study 1 and Study 2 results

To synthesize results across studies, we conducted mega-analyses (i.e., combining data from Study 1 and Study 2 into a combined dataset with a dummy code for study and then refitting the same models as in individual studies; *n*_total_ = 19,822).

In mega-analytic simultaneous regression models controlling for PSAT scores, gender, fee waiver status, and ethnicity, strategic self-control predicted practice time (*B* = 0.64, *β* = .33, *p* < .001, 95% CI [0.61, 0.67]) better than willpower (*B* = 0.07, *β* = .05, *p* < .001, 95% CI [0.04, 0.09], linear contrast: *B* = 0.57, 95% CI [0.53, 0.61], *t*(13831) = 30.88, *p* < .001). Strategic self-control also predicted SAT scores (*B* = 11.66, *β* = .02, *p* < .001, 95% CI [8.22, 15.11]) better than willpower (*B* = -0.69, *β* = < .01, *p* = .610, 95% CI [-3.36, 1.98]), linear contrast: *B* = 12.36, 95% CI [7.88, 16.84], *t*(14733) = 5.41, *p* < .001).

Mega-analytic mediation analyses showed that the number of self-control strategies used predicted greater practice time (*B* = 0.24, *β* = .54, *p* < .001, 95% CI [0.22, 0.26]) and SAT scores (*B* = 4.17, *β* = .05, *p* < .001, 95% CI [2.55, 5.78]), with declining marginal returns (practice time: *B* = -0.02, *β* = -.27, *p* < .001, 95% CI [-0.02, -0.02]; SAT scores: *B* = -0.33, *β* = -.03, *p* < .01, 95% CI [-0.56, -0.10]), and the benefits of strategic self-control were mediated by practice time (average indirect effect: *B* = 6.86, 95% CI [6.28, 7.47]).

## General discussion

In two national field studies involving nearly 20,000 adolescents, we examined strategic self-control and willpower in the context of the SAT college entrance exam. Results were consistent across Study 1 and a preregistered replication in Study 2.

Across both studies, compared with relying on willpower, using at least one self-control strategy predicted more time spent practicing for the SAT and higher SAT scores when controlling for demographics and prior achievement. In addition, across both studies, we observed a decelerating dose-response relationship between the number of self-control strategies used and total practice time, and this in turn mediated the relationship between self-control strategies and SAT scores. In other words, using more (versus fewer) self-control strategies appeared beneficial, but there were diminishing *marginal* benefits of using additional self-control strategies.

In contrast, we found that willpower had inconsistent benefits. Specifically, when controlling for strategic self-control, willpower showed modest positive benefits in Study 1, but in Study 2, using a refined questionnaire measure to explicitly exclude the use of self-control strategies (e.g., “I didn’t use different ways to practice, I just forced myself to do it”), showed negative effects on both practice time and SAT performance. Pooling data from Study 1 and Study 2, our mega-analytic estimates suggest a weak positive relationship between willpower and practice and no relationship with SAT scores.

How do these findings square with prior research? As noted earlier, the collective findings of one-week ESM studies are equivocal regarding the relative benefits of strategic self-control and willpower [21,22]. Clearly, it is sometimes necessary to directly modulate responses when goals conflict, and the deployment of self-control strategies is not entirely effortless [32]. Nevertheless, we conjecture that when pursuing real-world goals over extended time frames—studying in the month before the SAT exam, for example—a diverse repertoire of strategies targeting the antecedents of goal conflict constitutes a more efficient and sustainable approach. Consistent with this view, in both our samples, we observed a dose-response relationship between the number of different self-control strategies deployed and both practice behavior and SAT performance. That said, the declining marginal returns observed across both studies suggests that although more is better when it comes to strategies, one’s toolbox of self-control strategies need not be infinitely large. This finding is particularly notable as it contrasts with past work that showed a linear benefit of using multiple strategies [22]. More research is needed to clarify why we observe decreasing benefits of additional strategies, perhaps taking inspiration from other fields, such as pharmacology, where there are similarly shaped dose-response curves.

Several limitations of the current investigation speak to inherent tradeoffs between tightly controlled laboratory studies and large-scale field studies. First, our measures of self-control approaches and practice time were administered retrospectively by the College Board within two weeks of students taking the SAT. Relatedly, to minimize survey time, questionnaire measures were brief. For example, in Study 2, students saw only 5 out of 17 strategic self-control and willpower items. This reduction in the number of possible choices may have contributed to smaller effects in Study 2 than Study 1. Future prospective longitudinal studies employing open-ended questions and experience sampling are needed to confirm and enrich the broad-stroke findings we report here. Likewise, in future research, it may be possible to develop more symmetric measures of strategic self-control and willpower since, in the present investigation, the latter were longer and more specific than the former.

The external validity of our investigation is limited by the representativeness of the adolescents who voluntarily opted to participate. As a group, participants scored above-average on the PSAT exam. Establishing the generalizability of our conclusions will require research with students of more diverse achievement levels as well as older and younger students—and indeed studies that explore self-control across a wider array of life domains.

Finally, the correlational nature of these studies precludes causal inferences. Stronger claims for the efficacy of strategic self-control and willpower require experimental manipulation. One field experiment, for instance, found that students who were assigned to use a self-control strategy rather than willpower made more progress toward their goals [32]. For both theoretical and practical reasons, therefore, we hope to see more random-assignment field experiments comparing willpower with a variety of situational and cognitive self-control strategies [see 25].

In this investigation, we found that using at least one self-control strategy predicts an SAT score increase of between 7 and 19 points. By comparison, formal coaching for the SAT, which can cost hundreds or thousands of dollars, is associated with an increase of between 13 and 26 points on the SAT [33–35]. The relationship between strategic self-control and SAT scores, though small by conventional standards [36], are comparable to those observed in educational interventions [37]. Given the range of life outcomes that depend on self-control [24,38] and the increasingly wide availability of educational resources (e.g., Khan Academy), it may be especially cost-effective to empower students to approach self-control more strategically. Possibilities include school curricula in which students learn and practice diverse approaches to self-control [see 3,39], as well as creative collaborations, such as self-regulation skill-building episodes (e.g., Cookie Monster modeling the strategy of looking away from cookies to resist eating them) in Sesame Street programming [40]. Since strategic self-control is also associated with subjective well-being [41], such psychoeducational interventions are an especially promising direction for helping young people thrive [42].

On a theoretical level, our findings underscore the importance of distinguishing between more versus less strategic approaches to self-control. This distinction may not be obvious to the lay audience. Indeed, when asked what is important “when working to make a behavior or lifestyle change,” American adults rate “being able to resist temptations” as more important than more strategic approaches, including “monitoring my progress toward my goals” and “avoiding the people, things, or situations that lead to temptations” [1]. It may be that individuals misinterpret the sensation of effortfully forcing themselves to adhere to goals as valuable [43]. And it has been speculated that fatigue associated with directly resisting temptation reinforces the mindset that self-control runs out with use [44,45].

The present investigation makes clear that we need a better understanding of when and how individuals should rely on willpower versus more strategic forms of self-control. Nobel Laureate economist Thomas Schelling [46] once pointed out that getting what we want often entails artful “tricks we play on ourselves to make us do the things we ought to do or to keep us from the things we ought to foreswear” (p. 290). More recently, Job and colleagues [47] have called for “information about effective strategies that can help people avoid self-regulatory failures” (p. 646). We look forward to more research, both in the laboratory and in the field, on strategic self-control and polyregulation.

## Supporting information

S1 Appendix Correlations, descriptive statistics, and additional analyses for Studies 1 and 2. (DOCX)
