# Supplementary material for: Self-control and SAT outcomes: Evidence from two national field studies
Source: PLoS One. 2022 Sep 28;17(9):e0274380. doi: 10.1371/journal.pone.0274380 (PMC9518863; doi:10.1371/journal.pone.0274380)
Supplement: S1 Appendix — (DOCX) [file pone.0274380.s001.docx]

**S1 Online Appendix**

Correlations and Descriptive Statistics for Studies 1 and 2

Robustness Checks: Alternate Models in Studies 1 and 2

Exploratory Analyses of Individual Strategies in Studies 1 and 2

**Correlations and Descriptive Statistics**

As shown in Table S1, we calculated bivariate correlations and descriptive statistics for each of the variables in Studies 1 and 2.

| **Table S1. Correlations and Descriptive Statistics for Studies 1 and 2** | | | | | | | | | | | | | | |  | | |  | | |  | | |  | | |  | | |  | | |  |  |
| --- | --- | --- | --- | --- | --- | --- | --- | --- | --- | --- | --- | --- | --- | --- | --- | --- | --- | --- | --- | --- | --- | --- | --- | --- | --- | --- | --- | --- | --- | --- | --- | --- | --- | --- |
| Study 1 | | | | | | | | | | | | | | |  | | |  | | |  | | |  | | |  | | |  | | |  |  |
| Variable | | 1 | | 2 | | 3 | | 4 | | 5 | | 6 | | 7 | | | 8 | | | 9 | | | 10 | | | 11 | | | 12 | | | 13 | | |
| 1. Number of Self-Control Strategies | |  | |  | |  | |  | |  | |  | |  | | |  | | |  | | |  | | |  | | |  | | |  | | |
| 2. At least one Self-Control Strategy | | .58*** | |  | |  | |  | |  | |  | |  | | |  | | |  | | |  | | |  | | |  | | |  | | |
| 3. Willpower | | .13*** | | .24*** | |  | |  | |  | |  | |  | | |  | | |  | | |  | | |  | | |  | | |  | | |
| 4. Practice Time | | .46*** | | .49*** | | .26*** | |  | |  | |  | |  | | |  | | |  | | |  | | |  | | |  | | |  | | |
| 5. SAT Score | | .05*** | | .06*** | | .09*** | | .15*** | |  | |  | |  | | |  | | |  | | |  | | |  | | |  | | |  | | |
| 6. PSAT Score | | < .01 | | .02 | | .07*** | | .07*** | | .89*** | |  | |  | | |  | | |  | | |  | | |  | | |  | | |  | | |
| 7. Female | | .06*** | | .03* | | .06*** | | -.03* | | -.18*** | | -.15*** | |  | | |  | | |  | | |  | | |  | | |  | | |  | | |
| 8. Fee waiver | | .01 | | .02 | | -.02† | | -.01 | | -.22*** | | -.24*** | | .03* | | |  | | |  | | |  | | |  | | |  | | |  | | |
| 9. White | | -.09*** | | -.07*** | | -.01 | | -.17*** | | .01 | | .01 | | .01 | | | -.23*** | | |  | | |  | | |  | | |  | | |  | | |
| 10. Asian | | .08*** | | .09*** | | .04** | | .24*** | | .27*** | | .26*** | | -.07*** | | | < .01 | | | -.52*** | | |  | | |  | | |  | | |  | | |
| 11. Hispanic | | - .01 | | -.04** | | -.04** | | -.06*** | | -.17*** | | -.16*** | | .03† | | | .19*** | | | -.40*** | | | -.20*** | | |  | | |  | | |  | | |
| 12. African American | | .03† | | .02 | | .01 | | .01 | | -.21*** | | -.21*** | | .06*** | | | .19*** | | | -.32*** | | | -.16*** | | | -.12*** | | |  | | |  | | |
| 13. Other Ethnicity | | .01 | | .02 | | < .01 | | < .01 | | -.02† | | -.02 | | .02 | | | < .01 | | | -.22*** | | | -.11*** | | | -.08*** | | | -.07*** | | |  | | |
| *M* | | 3.08 | | 82.78% | | 72.35% | | 2.56 | | 1,252 | | 1,174 | | 69.66% | | | 12.62% | | | 51.07% | | | 20.49% | | | 13.09% | | | 9.02% | | | 4.24% | | |
| *SD* | | 2.40 | |  | |  | | .86 | | 176 | | 179 | |  | | |  | | |  | | |  | | |  | | |  | | |  | | |
| *n* | | 5,563 | | 5,563 | | 5,563 | | 5,033 | | 5,550 | | 5,563 | | 5,563 | | | 5,563 | | | 5,563 | | | 5,563 | | | 5,563 | | | 5,563 | | | 5,563 | | |
| Study 2 | | | | | | | | | | | | | | | | | | | | | | | | | | | | | | | | | |  |
| Variable | 1 | | 2 | | 3 | | 4 | | 5 | | 6 | | 7 | | | 8 | | | 9 | | | 10 | | | 11 | | | 12 | | | 13 | | |  |
| 1. Number of Self-Control Strategies |  | |  | |  | |  | |  | |  | |  | | |  | | |  | | |  | | |  | | |  | | |  | | |  |
| 2. At least one Self-Control Strategy | .63*** | |  | |  | |  | |  | |  | |  | | |  | | |  | | |  | | |  | | |  | | |  | | |  |
| 3. Willpower | -.15*** | | -.04*** | |  | |  | |  | |  | |  | | |  | | |  | | |  | | |  | | |  | | |  | | |  |
| 4. Practice Time | .27*** | | .19*** | | -.10*** | |  | |  | |  | |  | | |  | | |  | | |  | | |  | | |  | | |  | | |  |
| 5. SAT Score | -.06*** | | -.03*** | | -.01 | | .22*** | |  | |  | |  | | |  | | |  | | |  | | |  | | |  | | |  | | |  |
| 6. PSAT Score | -.09*** | | -.06*** | | < .01 | | .10*** | | .89*** | |  | |  | | |  | | |  | | |  | | |  | | |  | | |  | | |  |
| 7. Female | .06*** | | .08*** | | < .01 | | -.04*** | | -.19*** | | -.15*** | |  | | |  | | |  | | |  | | |  | | |  | | |  | | |  |
| 8. Fee waiver | .03*** | | .03*** | | -.01 | | -.03*** | | -.10*** | | -.10*** | | .04*** | | |  | | |  | | |  | | |  | | |  | | |  | | |  |
| 9. White | -.04*** | | -.03*** | | -.01 | | -.16*** | | -.02* | | .02* | | .03*** | | | -.16*** | | |  | | |  | | |  | | |  | | |  | | |  |
| 10. Asian | .02** | | <.01 | | < .01 | | .27*** | | .35*** | | .28*** | | -.11*** | | | .01 | | | -.50*** | | |  | | |  | | |  | | |  | | |  |
| 11. Hispanic | .01 | | .03** | | .02* | | -.10*** | | -.25*** | | -.22*** | | .05*** | | | .19*** | | | -.37*** | | | -.27*** | | |  | | |  | | |  | | |  |
| 12. African American | .03** | | .01 | | -.02 | | .01 | | -.21*** | | -.21*** | | .06*** | | | .05*** | | | -.25*** | | | -.18*** | | | -.14*** | | |  | | |  | | |  |
| 13. Other Ethnicity | -.01 | | <.01 | | .01 | | -.03*** | | < .01 | | < .01 | | .02* | | | -.03** | | | -.19*** | | | -.14*** | | | -.11*** | | | -.07*** | | |  | | |  |
| *M* | 2.01 | | 85.97% | | 50.73% | | 2.80 | | 1,254 | | 1,164 | | 64.04% | | | 7.64% | | | 40.57% | | | 26.59% | | | 16.71% | | | 8.45% | | | 5.22% | | |  |
| *SD* | 1.29 | |  | |  | | 0.54 | | 178 | | 169 | |  | | |  | | |  | | |  | | |  | | |  | | |  | | |  |
| *n* | 14,259 | | 14,250 | | 9,278 | | 13,549 | | 14,138 | | 14,259 | | 14,259 | | | 14,259 | | | 14,259 | | | 14,259 | | | 14,259 | | | 14,259 | | | 14,259 | | |  |
| *Note.* Coefficients represent bivariate correlations. Means (*M*), standard deviations (*SD*), and sample size (*n*) for each variable are indicated below bivariate correlations. * *p* < .05, ** *p* < .01, *** *p* < .001. | | | | | | | | | | | | | | | | | | | | | | | | | | | | | | | | | |  |

## **Robustness Checks**

### **Alternate models in Studies 1 and 2**

We ran a series of robustness checks beyond what is reported in the main text and pre-registered analyses, which are reported in Tables S2, S3, and S4. Results remain consistent with the main text regardless of how we analyze the data. First, in Table S2, we present the results of our pre-registered analyses: Models 1 and 2 are the binary comparisons between strategic self-control and willpower presented as the first analyses in the main text. Models 3, 4, and 5 show that the number of self-control strategies used predicts practice time, SAT scores, and the mediation of SAT scores through practice time. These models do not differ from the results presented in the main text except that they do not include a quadratic term. Models 6, 7, and 8 represent these same models with the addition of a quadratic term, as presented in the main text. The results do not differ between either set of analyses. Models 9, 10, and 11 represent the binary strategic self-control variable as the primary predictor in the mediation analyses, presented here as a robustness check.

In Table S3, we present the results of hierarchical regression models assessed as a robustness check for our preregistered linear contrasts comparing the effects of strategic self-control and willpower on SAT scores and practice time. For the regression models for each outcome, Model 1 includes covariates, Model 2 adds PSAT, Model 3 adds willpower, and finally, Model 4 adds whether students used at least one self-control strategy. For models predicting SAT scores across studies, each step significantly improved the predictive value of the model. For models predicting practice time across studies, steps adding willpower and the use of at least one self-control strategy significantly improved the model, but the step adding PSAT did not. These results are in line with the results of the linear contrast reported in the manuscript.

In Table S4, we present models comparing willpower and strategic self-control in Study 2 for only students who saw two or more willpower items and three or fewer strategic self-control items. The purpose of these analyses was to assess whether the results of our models reported in the main manuscript would change for students who saw a similar number of willpower and strategic self-control items. In Model 1, binary measures of willpower and strategic self-control, controlling for covariates, predict log practice time. In Model 2, the same variables predict SAT scores. For both log practice time and SAT scores, strategic self-control predicted the outcome better than willpower, and willpower had a small, marginally significant negative effect. These results are in line with the main results reported in the manuscript.

**Table S2. Alternate Models in Studies 1 and 2 Predicting Practice Time and SAT Scores**

| Study 1 |  |  |  |  |  |  |  |  |  |  |  |
| --- | --- | --- | --- | --- | --- | --- | --- | --- | --- | --- | --- |
| Model | 1 | 2 | 3 | 4 | 5 | 6 | 7 | 8 | 9 | 10 | 11 |
| Outcome | Practice | SAT | SAT | Practice | SAT | SAT | Practice | SAT | SAT | Practice | SAT |
| Willpower | 0.15*** (0.01) | 0.02** (0.01) |  |  |  |  |  |  |  |  |  |
| Self-Control Strategies |  |  |  |  |  |  |  |  |  |  |  |
| Binary Strategic Self-Control | 0.44*** (0.01) | 0.04*** (0.01) |  |  |  |  |  |  | 0.05*** (0.01) | 0.48*** (0.01) | 0.01* (0.01) |
| Number of Self-Control Strategies |  |  | 0.05*** (0.01) | 0.44*** (0.01) | 0.02** (0.01) | 0.10*** (0.02) | 0.95*** (0.03) | 0.03†  (0.02) |  |  |  |
| Number of Self-Control Strategies^2^ |  |  |  |  |  | -0.05** (0.02) | -0.54*** (0.03) | -0.01  (0.02) |  |  |  |
| Practice time |  |  |  |  | 0.07*** (0.01) |  |  | 0.07*** (0.01) |  |  | 0.07*** (0.01) |
| Covariates |  |  |  |  |  |  |  |  |  |  |  |
| PSAT score | 0.01 (0.01) | 0.87*** (0.01) | 0.86*** (0.01) | 0.02†  (0.01) | 0.86*** (0.01) | 0.86*** (0.01) | 0.02  (0.01) | 0.86*** (0.01) | 0.86*** (0.01) | 0.02  (0.01) | 0.86*** (0.01) |
| Female | -0.05*** (0.01) | -0.04*** (0.01) | -0.04*** (0.01) | -0.05*** (0.01) | -0.03*** (0.01) | -0.04*** (0.01) | -0.05*** (0.01) | -0.03*** (0.01) | -0.04*** (0.01) | -0.04** (0.01) | -0.03*** (0.01) |
| Fee waiver | -0.03* (0.01) | < 0.01 (0.01) | < 0.01 (0.01) | -0.02†  (0.01) | < 0.01 (0.01) | < 0.01 (0.01) | -0.03*  (0.01) | < 0.01 (0.01) | < 0.01 (0.01) | -0.03** (0.01) | < 0.01 (0.01) |
| Asian | 0.21*** (0.01) | 0.03*** (0.01) | 0.03*** (0.01) | 0.21*** (0.01) | 0.02** (0.01) | 0.03*** (0.01) | 0.20*** (0.01) | 0.02** (0.01) | 0.03*** (0.01) | 0.21*** (0.01) | 0.02** (0.01) |
| African American | 0.05*** (0.01) | -0.03*** (0.01) | -0.03*** (0.01) | 0.05*** (0.01) | -0.03*** (0.01) | -0.03*** (0.01) | 0.04*** (0.01) | -0.03*** (0.01) | -0.03*** (0.01) | 0.05*** (0.01) | -0.03*** (0.01) |
| Hispanic | 0.02 (0.01) | -0.03*** (0.01) | -0.03*** (0.01) | < 0.01 (0.01) | -0.02*** (0.01) | -0.02** (0.01) | 0.01  (0.01) | -0.02** (0.01) | -0.02** (0.01) | 0.02  (0.01) | -0.02** (0.01) |
| Other ethnicity | 0.03* (0.01) | -0.01  (0.01) | -0.01  (0.01) | 0.02†  (0.01) | -0.01  (0.01) | -0.01  (0.01) | 0.02*  (0.01) | -0.01  (0.01) | -0.01  (0.01) | 0.03* (0.01) | -0.01  (0.01) |
| *R^2^* | .31 | .80 | .80 | .26 | .81 | .80 | .30 | .81 | .80 | .29 | .81 |
| *n* | 5032 | 5549 | 5022 | 5022 | 5022 | 5022 | 5022 | 5022 | 5022 | 5022 | 5022 |
| Study 2 |  |  |  |  |  |  |  |  |  |  |  |
| Model | 1 | 2 | 3 | 4 | 5 | 6 | 7 | 8 | 9 | 10 | 11 |
| Outcome | Practice | SAT | SAT | Practice | SAT | SAT | Practice | SAT | SAT | Practice | SAT |
| Willpower | -0.08*** (0.01) | -0.01**  (< 0.01) |  |  |  |  |  |  |  |  |  |
| Self-Control Strategies |  |  |  |  |  |  |  |  |  |  |  |
| Binary Strategic Self-Control | 0.20*** (0.01) | 0.02**  (< 0.01) |  |  |  |  |  |  | 0.01**  (< 0.01) | 0.17*** (0.01) | -0.02**  (< 0.01) |
| Number of Self-Control Strategies |  |  | 0.01**  (< 0.01) | 0.22*** (0.01) | -0.02***  (< 0.01) | 0.03* (0.01) | 0.34*** (0.02) | -0.02† (0.01) |  |  |  |
| Number of Self-Control Strategies^2^ |  |  |  |  |  | -0.02  (0.01) | -0.12*** (0.02) | < 0.01  (0.01) |  |  |  |
| Practice time |  |  |  |  | 0.16***  (< 0.01) |  |  | 0.16***  (< 0.01) |  |  | 0.15***  (< 0.01) |
| Covariates |  |  |  |  |  |  |  |  |  |  |  |
| PSAT score | 0.04*** (0.01) | 0.84***  (< 0.01) | 0.83***  (< 0.01) | 0.04*** (0.01) | 0.83***  (< 0.01) | 0.83***  (< 0.01) | 0.04*** (0.01) | 0.83***  (< 0.01) | 0.83***  (< 0.01) | 0.02*** (0.01) | 0.83***  (< 0.01) |
| Female | -0.02* (0.01) | -0.05***  (< 0.01) | -0.05***  (< 0.01) | -0.02**  (0.01) | -0.05***  (< 0.01) | -0.05***  (< 0.01) | -0.02***  (0.01) | -0.05***  (< 0.01) | -0.05***  (< 0.01) | -0.02** (0.01) | -0.05***  (< 0.01) |
| Fee waiver | -0.02*** (0.01) | -0.01*  (< 0.01) | -0.01†  (< 0.01) | -0.03*** (0.01) | < 0.01  (< 0.01) | -0.01†  (< 0.01) | -0.03*** (0.01) | < 0.01  (< 0.01) | -0.01†  (< 0.01) | -0.03*** (0.01) | < 0.01  (< 0.01) |
| Asian | 0.27*** (0.01) | 0.09*** (0.01) | 0.09***  (< 0.01) | 0.20*** (0.01) | 0.06***  (< 0.01) | 0.09***  (< 0.01) | 0.20*** (0.01) | 0.06***  (< 0.01) | 0.09***  (< 0.01) | 0.21*** (0.01) | 0.06***  (< 0.01) |
| African American | 0.04*** (0.01) | -0.02** (0.01) | -0.02***  (< 0.01) | 0.03*** (0.01) | -0.03***  (< 0.01) | -0.02***  (< 0.01) | 0.03*** (0.01) | -0.03***  (< 0.01) | -0.02***  (< 0.01) | 0.04*** (0.01) | -0.03***  (< 0.01) |
| Hispanic | -0.02* (0.01) | -0.04*** (0.01) | -0.04***  (< 0.01) | -0.01  (0.01) | -0.04***  (< 0.01) | -0.04***  (< 0.01) | -0.01  (0.01) | -0.04***  (< 0.01) | -0.04***  (< 0.01) | -0.01†  (0.01) | -0.04***  (< 0.01) |
| Other ethnicity | <0.01 (0.01) | < 0.01  (< 0.01) | < 0.01  (< 0.01) | <0.01  (0.01) | < 0.01  (< 0.01) | < 0.01  (< 0.01) | < 0.01  (0.01) | < 0.01  (< 0.01) | < 0.01  (< 0.01) | < 0.01  (0.01) | < 0.01  (< 0.01) |
| *R^2^* | .13 | .81 | .80 | .15 | .82 | .80 | .16 | .82 | .81 | .12 | .82 |
| *n* | 8808 | 9193 | 13431 | 13431 | 13431 | 13431 | 13431 | 13431 | 13431 | 13431 | 13431 |
| *Note:* Coefficients represent standardized betas for each regression. Standard errors are indicated in parentheses. † *p* < .10; * *p* < .05, ** *p* < .01, *** *p* < .001.   \| **Table S3. Hierarchical Regression to Assess the Effect of the Use of Any Self-Control Strategy Beyond Willpower, PSAT, and Covariates on Outcomes** \| \| \| \| \| \| \| \| \| \| \| \| --- \| --- \| --- \| --- \| --- \| --- \| --- \| --- \| --- \| --- \| --- \| \| Study 1 \| \| \| \| \| \| \| \| \| \| \| \|  \|  \| SAT \| \| \| \|  \| Practice \| \| \| \| \|  \| \| Model 1 \| Model 2 \| Model 3 \| Model 4 \|  \| Model 1 \| Model 2 \| Model 3 \| Model 4 \| \| Female \| \| -.14† \| -.03** \| -.04** \| -.04** \|  \| -.02 \| -.02 \| -.04 \| -.05 \| \| Fee waiver \| \| -.16*** \| < .01 \| < .01 \| < .01 \|  \| -.03† \| -.02 \| -.02 \| -.03* \| \| *Ethnicity* \| \|  \|  \|  \|  \|  \|  \|  \|  \|  \| \|  \| Asian \| .21*** \| .04*** \| .04*** \| .03*** \|  \| .26*** \| .25*** \| .25*** \| .21*** \| \|  \| Black \| -.15*** \| -.03*** \| -.03*** \| -.03*** \|  \| .06*** \| .06*** \| .06*** \| .05*** \| \|  \| Hispanic \| -.11*** \| -.03*** \| -.03*** \| -.03*** \|  \| .01 \| .01 \| .01 \| .02† \| \|  \| Other \| -.02 \| -.01 \| -.01 \| -.01 \|  \| .03* \| .03* \| .03* \| .03* \| \| PSAT \| \|  \| .86*** \| .86*** \| .86*** \|  \|  \| .02 \| -.01 \| .01 \| \| Willpower \| \|  \|  \| .03*** \| .02** \|  \|  \|  \| .25*** \| .15*** \| \| Strategic Self-Control \| \|  \|  \|  \| .04*** \|  \|  \|  \|  \| .45*** \| \| *n* \| \| 5550 \| 5550 \| 5550 \| 5550 \|  \| 5022 \| 5022 \| 5022 \| 5022 \| \| *R*^2^ \| \| .1723 \| .8017 \| .8025 \| .8041 \|  \| .0636 \| .0638 \| .1269 \| .3075 \| \| *R*^2^ change \| \|  \| .629*** \| .001*** \| .002*** \|  \|  \| < .01 \| .063*** \| .181*** \| \| Study 2 \| \| \| \| \| \| \| \| \| \| \| \|  \|  \| SAT \| \| \| \|  \| Practice \| \| \| \| \|  \| \| Model 1 \| Model 2 \| Model 3 \| Model 4 \|  \| Model 1 \| Model 2 \| Model 3 \| Model 4 \| \| Female \| \| -.15† \| -.05* \| -.05* \| -.05* \|  \| -.01*** \| <.01 \| <.01 \| -.02*** \| \| Fee waiver \| \| -.06*** \| -.01* \| -.01* \| -.01* \|  \| -.02* \| -.02* \| -.02* \| -.03** \| \| *Ethnicity* \| \|  \|  \|  \|  \|  \|  \|  \|  \|  \| \|  \| Asian \| .24*** \| .09*** \| .09*** \| .09*** \|  \| .21*** \| .21*** \| .21*** \| .20*** \| \|  \| Black \| -.17*** \| -.02** \| -.02** \| -.02** \|  \| .03** \| .03*** \| .03** \| .03*** \| \|  \| Hispanic \| -.20*** \| -.04*** \| -.04*** \| -.04*** \|  \| -.02* \| -.02† \| -.02† \| -.02* \| \|  \| Other \| -.01 \| < .01 \| < .01 \| < .01 \|  \| < .01 \| <.01 \| <.01 \| <.01 \| \| PSAT \| \|  \| .84*** \| .84*** \| .84*** \|  \|  \| .02* \| .02* \| .03*** \| \| Willpower \| \|  \|  \| -.01** \| -.01** \|  \|  \|  \| -.08** \| -.06*** \| \| Strategic Self-Control \| \|  \|  \|  \| .01** \|  \|  \|  \|  \| .16*** \| \| *n* \| \| 9194 \| 9194 \| 9194 \| 9194 \|  \| 8737 \| 8737 \| 8737 \| 8737 \| \| *R*^2^ \| \| .2079 \| .8045 \| .8047 \| .8050 \|  \| .0655 \| .0658 \| .0669 \| .1511 \| \| *R*^2^ change \| \|  \| .5966*** \| .0002** \| .0003** \|  \|  \| .0006* \| .0097*** \| .0397*** \| \| *Note*. Estimates for predictors are standardized beta coefficients. Stars for *R*^2^ change indicate significant *F* tests between models. † = *p* < .10; * = *p* < .05; ** = *p* < .01; *** = *p* < .001 \| \| \| \| \| \| \| \| \| \| \|   **Table S4. Main Models for Students in Study 2 Who Saw Two or More Willpower Items and Three or Fewer Strategic Self-Control Items**   \| Model \| Model 1 \| Model 2 \| \| --- \| --- \| --- \| \| Outcome \| Practice \| SAT \| \| Willpower \| -0.04†  (0.02) \| -0.03†  (0.02) \| \| Binary Strategic Self-Control \| 0.24***  (0.02) \| 0.10***  (0.02) \| \| Covariates \|  \|  \| \| PSAT score \| 0.01  (0.02) \| 0.85***  (0.01) \| \| Female \| -0.01  (0.02) \| -0.06***  (0.01) \| \| Fee waiver \| -0.06** (0.02) \| -0.01  (0.01) \| \| Asian \| 0.24*** (0.02) \| 0.09*** (0.01) \| \| African American \| 0.02  (0.02) \| -0.02* (0.01) \| \| Hispanic \| -0.02  (0.02) \| -0.05*** (0.01) \| \| Other ethnicity \| -0.02  (0.02) \| < 0.01  (0.01) \| \| *R^2^* \| .16 \| .81 \| \| *n* \| 2313 \| 2349 \| | | | | | | | | | | | |

## **Exploratory Analyses of Individual Strategies in Studies 1 and 2**

In exploratory analyses, we examined the frequency and predictive power of individual self-control strategies. In Table S5, we list self-control items and predictive validities from a series of regression models in which each individual strategy, controlling for all covariates, predicted SAT scores and practice time.

Across both studies, self-control strategies were effective at increasing practice time. All self-control strategies positively predicted practice time, with no strategies showing significant negative predictive power. Willpower, on the other hand, predicted practice time positively in Study 1, but negatively in Study 2. This may be the result of the wording changes in Study 2 to more directly contrast willpower and strategic self-control.

As might be expected, associations with the more distal outcome of SAT scores were more modest. In Study 1, 8 out of 12 self-control strategies positively predicted SAT score gains, while three approaches had no reliable association with SAT score gains. One item, “I thought about the skills I was building for later in life”, negatively correlated with SAT scores. In Study 2, five out of 14 approaches significantly positively, and three marginally positively, predicted SAT scores. As in Study 1, “I thought about the skills I was building for later in life” negatively correlated with SAT scores. Willpower items in Study 2 showed negative effects on both SAT scores and practice. Items that showed consistent benefits across both studies and outcomes tended to focus on planning strategies (such as setting a concrete study schedule), self-monitoring (such as tracking how often they studied), and changes to study environments that made it easier to focus (such as turning off their phone while studying).

**Table S5. Frequency and Effect Sizes of Individual Self-Control Strategies**

| Study 1 | | | |
| --- | --- | --- | --- |
| Willpower item | % Used | SAT 𝛽 | Practice 𝛽 |
| I just forced myself to do it. | 72 | 0.03***  (0.01) | 0.25***  (0.01) |
| Strategic Self-Control items |  |  |  |
| I reminded myself why I was studying in the first place. | 59 | 0.04***  (0.01) | 0.32***  (0.01) |
| I chose to study in places that were easier to focus. | 49 | 0.03***  (0.01) | 0.27***  (0.01) |
| I set up a place to study that was free of distractions. | 40 | 0.04***  (0.01) | 0.28***  (0.01) |
| I disabled my phone while I practiced. | 31 | 0.03***  (0.01) | 0.21***  (0.01) |
| I set a concrete study schedule. | 24 | 0.05***  (0.01) | 0.30***  (0.01) |
| I tracked how often I studied. | 24 | 0.04***  (0.01) | 0.27***  (0.01) |
| I told my study goals to someone who cared about me. | 21 | 0.04***  (0.01) | 0.22***  (0.01) |
| I reminded myself that frustration is a sign of learning. | 19 | 0.02**  (0.01) | 0.16***  (0.01) |
| I thought about the skills I was building for later in life. | 16 | -0.01*  (0.01) | 0.14***  (0.01) |
| I made a visual reminder of why I was studying. | 12 | < 0.01  (0.01) | 0.15***  (0.01) |
| I tried to turn studying into a game. | 8 | 0.01  (0.01) | 0.09***  (0.01) |
| I made a study plan with a friend. | 6 | < 0.01  (0.01) | 0.07***  (0.01) |
|  |  |  |  |
| Study 2 | | | |
| Willpower items | % Used | SAT 𝛽 | Practice 𝛽 |
| I didn’t do anything in particular, I just willed myself to not get distracted. | 56 | -0.02*  (0.01) | -0.10**  (0.02) |
| I didn’t use different ways to practice, I just forced myself to do it. | 50 | -0.02**  (0.01) | -0.11***  (0.02) |
| I didn’t use strategies, I just gritted my teeth and tried hard to study. | 32 | -0.01  (0.01) | -0.09**  (0.02) |
| Strategic Self-Control items |  |  |  |
| I chose to study in places that were easier to focus. | 77 | 0.01  (0.01) | 0.15***  (0.02) |
| I reminded myself why I was studying in the first place. | 76 | <0.01  (0.01) | 0.14***  (0.02) |
| I turned my attention away from distractions while I studied. | 73 | 0.03**  (0.01) | 0.18***  (0.02) |
| I set up a place to study that was free of distractions. | 65 | 0.01†  (0.01) | 0.24***  (0.02) |
| I turned off or hid my phone while I practiced. | 54 | 0.02**  (0.01) | 0.22***  (0.02) |
| I changed the way I was thinking about the SAT to make it easier to study. | 53 | 0.01†  (0.01) | 0.15***  (0.02) |
| I thought of the ways that distractions from practicing would be harmful. | 51 | 0.02**  (0.01) | 0.15***  (0.02) |
| I tracked how often I studied. | 46 | 0.05***  (0.01) | 0.30***  (0.02) |
| I told my study goals to someone who cared about me. | 44 | 0.01†  (0.01) | 0.26***  (0.02) |
| I set reminders to practice somewhere I would see them. | 43 | <0.01  (0.01) | 0.16***  (0.02) |
| I set a concrete study schedule somewhere I would see regularly. | 40 | 0.07***  (0.01) | 0.35***  (0.02) |
| I thought about the skills I was building for later in life. | 38 | -0.02**  (0.01) | 0.11***  (0.02) |
| I reminded myself that frustration is a sign of learning. | 35 | 0.01  (0.01) | 0.10***  (0.02) |
| I tried to turn studying into a game. | 17 | -0.01  (0.01) | 0.07***  (0.02) |

*Note.* Standardized coefficients are from separate linear regression models predictive practice time and SAT score, respectively, controlling for PSAT score, fee waiver status, gender, and ethnicity. Standard errors are indicated in parentheses. † *p* < .10; * *p* < .05, ** *p* < .01, *** *p* < .001.
